# GUAVA: A Graphical User Interface for the Analysis and Visualization of ATAC-seq Data

**DOI:** 10.3389/fgene.2018.00250

**Published:** 2018-07-17

**Authors:** Mayur Divate, Edwin Cheung

**Affiliations:** Faculty of Health Sciences, University of Macau, Macau, Macau

**Keywords:** ATAC-seq data analysis, GUI, bioinformatic tool, ATAC-seq, NGS data analysis

## Abstract

Assay for Transposase Accessible Chromatin with high-throughput sequencing (ATAC-seq) is a powerful genomic technology that is used for the global mapping and analysis of open chromatin regions. However, for users to process and analyze such data they either have to use a number of complicated bioinformatic tools or attempt to use the currently available ATAC-seq analysis software, which are not very user friendly and lack visualization of the ATAC-seq results. Because of these issues, biologists with minimal bioinformatics background who wish to process and analyze their own ATAC-seq data by themselves will find these tasks difficult and ultimately will need to seek help from bioinformatics experts. Moreover, none of the available tools provide complete solution for ATAC-seq data analysis. Therefore, to enable non-programming researchers to analyze ATAC-seq data on their own, we developed a tool called Graphical User interface for the Analysis and Visualization of ATAC-seq data (GUAVA). GUAVA is a standalone software that provides users with a seamless solution from beginning to end including adapter trimming, read mapping, the identification and differential analysis of ATAC-seq peaks, functional annotation, and the visualization of ATAC-seq results. We believe GUAVA will be a highly useful and time-saving tool for analyzing ATAC-seq data for biologists with minimal or no bioinformatics background. Since GUAVA can also operate through command-line, it can easily be integrated into existing pipelines, thus providing flexibility to users with computational experience.

## Introduction

Eukaryotic DNA is packaged together with histones to form nucleosomes, the basic unit of chromatin, which is important in determining the structure of the genome and the regulation of biological processes such as transcription. The regulatory information within chromatin can be uncovered through high-throughput sequencing methods for assaying chromatin accessibility, nucleosome positioning, histone modifications, and transcription factor (TF) occupancy. Assay for Transposase Accessible Chromatin with high-throughput sequencing (ATAC-seq) is a simple, yet highly robust and sensitive genome-wide technique that was recently developed for identifying nucleosome-bound and nucleosome-free regions of chromatin as well as TF occupancy (Buenrostro et al., 2013). Briefly, ATAC-seq accomplishes this by simultaneously fragmenting and tagging genomic DNA with sequencing adaptors using the hyperactive Tn5 transposase enzyme (Buenrostro et al., 2013). In contrast to similar technologies such as DNase I hypersensitive sites sequencing (DNase-seq) (Song and Crawford, 2010), ATAC-seq requires less cells (Buenrostro et al., 2013), meaning that ATAC-seq can be applied in many more experimental situations. In comparison to formaldehyde-assisted isolation of regulatory elements with sequencing (FAIRE-seq) (Giresi and Lieb, 2009), another global chromatin accessibility technology, ATAC-seq data has a lower background and therefore better signal-to-noise ratio (Buenrostro et al., 2013). Finally, ATAC-seq, unlike DNase-seq and FAIRE-seq, is much simpler and does not involve loss-prone steps like adapter ligation, gel purification, and crosslink reversal (Buenrostro et al., 2013). Therefore, due to its ease of use, speed, low starting material requirement, reliability and multiplexing potential, ATAC-seq has been widely adopted and will most likely be the preferred method of choice in comparison to FAIRE-seq and DNase-seq for global chromatin accessibility analysis.

Currently, several bioinformatic tools are available for processing or analyzing ATAC-seq data including, NucleoATAC (Schep et al., 2015), ATACseqQC (Ou et al., 2018), ATAC-Seq/DNase-Seq Pipeline^1^, and I-ATAC (Ahmed and Ucar, 2017). However, these tools are either command-line based or provide only partial solutions, but more importantly, most of them are complicated to use especially for biologists with little bioinformatics training, command line, and scripting experience. Moreover, these tools lack differential analysis, functional annotation, and visualization of ATAC-seq results (**Table 1**). Thus, we developed Graphical User interface for the Analysis and Visualization of ATAC-seq data (GUAVA), a simple user-friendly GUI-based application for processing, analyzing and visualizing ATAC-seq data. GUAVA accomplishes this by performing the following main tasks: (i) pre-process raw sequencing reads, (ii) map sequencing reads to a reference genome, (iii) filter and shift the mapped reads, (iv) identify and annotate the ATAC-seq peaks, (v) normalize and visualize the ATAC-seq data tracks, (vi) identify differentially enriched peaks, and (vii) identify over-represented gene ontology terms and pathways. GUAVA operates as a standalone program on computers with either Linux or Mac OS. To aid users, GUAVA package contains a complete reference manual.

**Table 1:** Comparison between GUAVA and available tools for ATAC-seq data analysis.

Features	GUAVA	NucleoATAC	I-ATAC	ATACseqQC	ATAC-Seq/DNase-Seq Pipeline
Graphical user interface	Yes	No	Yes	No	No
Adapter trimming	Yes	No	Yes	No	Yes
Alignment	Yes	No	Yes	No	Yes
Alignment filtering	Yes	No	Yes	No	Yes
Alignment shifting	Yes	Yes	Yes	Yes	Yes
Fragment size distribution plot	Yes	Yes	Yes	Yes	No
Peak calling	Yes	No	Yes	No	Yes
ATAC-seq differential analysis	Yes	No	No	No	No
Visualization of ATAC-seq data tracks	Yes	No	No	Yes	No
Annotation and function analysis	Yes	No	No	No	No
Nucleosome positing	No	Yes	No	Yes	No
Transcription factor foot printing	No	Yes	No	Yes	No

*A comparison between GUAVA and currently available tools for ATAC-seq data analysis based on features such read mapping, peak calling, and differential analysis, etc.*

## Materials and Methods

### Design and Implementation

**FIGURE 1:**
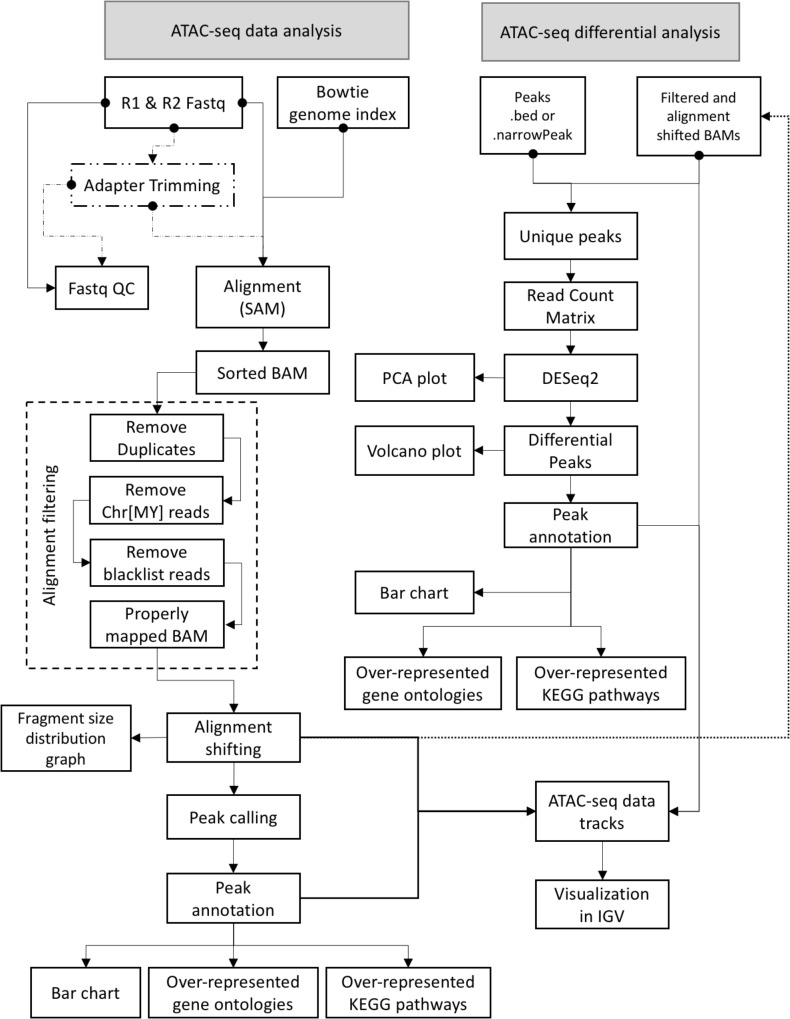
ATAC-seq data and differential analysis workflow for GUAVA. A diagram showing the GUAVA workflow for analyzing ATAC-seq data. The workflow has two parts (1) ATAC-seq data analysis: to process a ATAC-seq sample and (2) ATAC-seq differential analysis: to identify differentially enriched ATAC-seq signals between two conditions.

**FIGURE 2:**
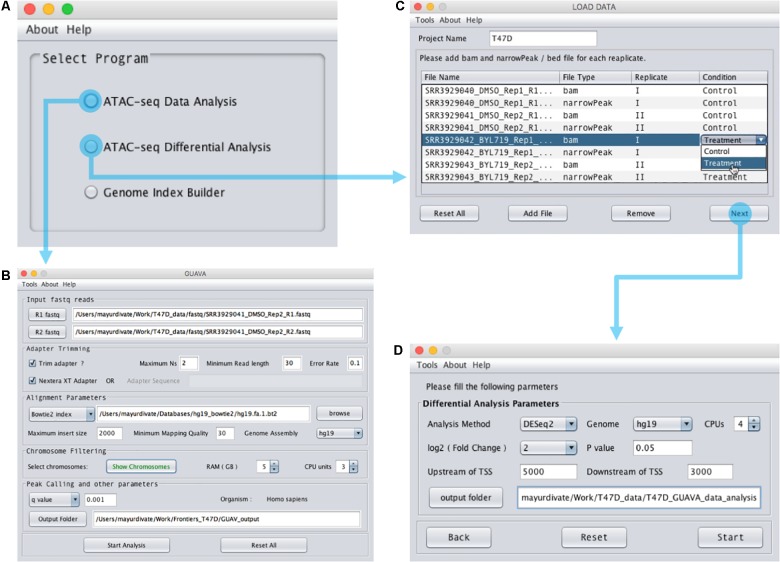
Design of the GUAVA input interface. **(A)** The main GUAVA window where users select the program they wish to use (ATAC-seq data analysis or ATAC-seq differential analysis). **(B)** Window for using ATAC-seq data analysis. To perform ATAC-seq data analysis, users load the sequencing data using the R1 fastq and R2 fastq buttons. Next, they choose between Bowtie and Bowtie2 from the drop-down menu in the “Alignment Parameters” section. If required, users can change the other parameters from the default value to another value. For example, the default RAM used for GUAVA is 1 GB but it can be changed if desired. Finally, to run the analysis users simply click the “Start Analysis” button. **(C)** Window for using ATAC-seq differential analysis. To perform ATAC-seq differential analysis, users set the project name, load the bam and bed files and then set the differential analysis parameters in **(D)** before selecting the “Start” button.

Graphical User interface for the Analysis and Visualization of ATAC-seq data is an open-source software that was implemented using Java programming language. GUAVA has been designed to be a fully automated software for analyzing and visualizing of ATAC-seq data. GUAVA depends on several different tools and methods such as Bowtie (Langmead et al., 2009) for mapping sequencing reads, MACS2 (Feng et al., 2012) for calling peaks, and DESeq2 (Love et al., 2014) for differential analysis. GUAVA contains two programs: (1) ATAC-seq data analysis (to process individual ATAC-seq samples or replicates) and (2) ATAC-seq differential analysis (to compare ATAC-seq samples from two different conditions). In addition, GUAVA has a genome index builder for non-programming researchers to create a genome index (Supplementary Figure S1). A detailed workflow for GUAVA is shown in **Figure 1**. The GUI to select the desired GUAVA program and upload the input files is shown in **Figure 2**.

### Part 1: ATAC-seq Data Analysis

**FIGURE 3:**
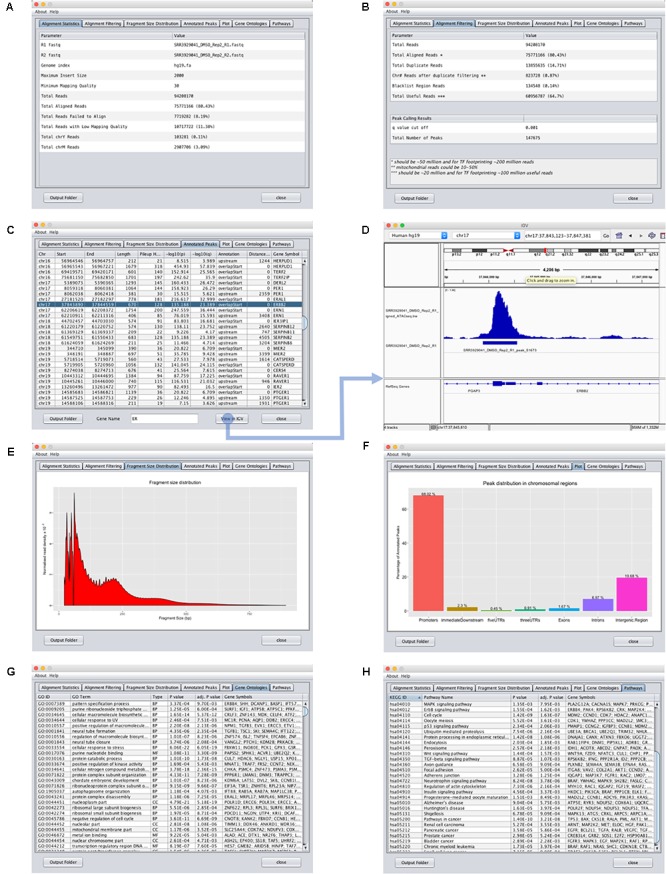
Output interface for GUAVA ATAC-seq data analysis. **(A)** Input summary and alignment statistics. **(B)** Read filtering and peak calling summary. **(C)** Peak annotation table with sorting and filtering functionality. Easy access to IGV for visualizing peaks and automatically generated normalized ATAC-seq signal by GUAVA. **(D)** Visualization of ATAC-seq peaks with IGV. **(E)** Graph showing the fragment size distribution. **(F)** Bar chart showing the percentage of peaks in various genomic locations such as the promoter, intron, exon, and UTR, etc. **(G)** Plot showing the percentage of the peaks upstream and downstream of the TSS of the nearest genes. *Different colors* indicate different ranges of distances from the TSS. **(H)** Over-represented KEGG pathways obtained using the ChIPpeakAnno bioconductor package.

To use the ATAC-seq data analysis program, the user simply needs to (1) upload the ATAC-seq paired-end fastq reads and (2) provide the Bowtie or Bowtie2 (Langmead and Salzberg, 2012) index reference genome using the input interface of the ATAC-seq data analysis program (**Figure 2B**). If the data has not been adapter trimmed, the user can select the trimming option. The user has the option to change several parameters such as the maximum insert size, the minimum mapping quality and the *p*/*q* value cut-off. Finally, after setting the output folder for storing the result files, the user can select “Start Analysis.” GUAVA will then automatically process and analyze the data as described below. Once GUAVA has finished the analysis, results such as alignment statistics, fragment size distribution, ATAC-seq peaks, and their functional annotations are shown in the output interface (**Figure 3**). The user can also find an excel file containing the same results in the output folder (Supplementary Data Sheet S1).

#### Pre-processing of the Raw Reads

In many instances, the raw sequenced reads used for ATAC-seq analysis will contain adapter sequences. Therefore, before mapping the raw reads to the genome, the adapter sequences should be trimmed off, otherwise any reads with adapter sequences will fail to map. For adapter trimming, GUAVA uses cutadapt (Martin, 2011). If adapter sequences have already been trimmed, this step can be omitted. After adapter trimming, the reads will be saved in fastq format. GUAVA will then generate a fastq quality report using the FastQC tool^2^. The FastQC report is stored inside the folder with the suffix FastQC_OUTPUT within the GUAVA output folder. In the FastQC report, “per base sequence quality” should be more than 20 for each nucleotide in the sequencing read and “per base N content” should not exceed 5%. If these minimum parameters are not met, this is an indication the sequencing reads are of poor quality. It should be noted that poor quality or sequencing errors often leads to low mapping rate and therefore, it is important to check the quality of the sequencing reads before mapping them to the genome.

#### Read Mapping

For mapping ATAC-seq reads to the genome, the user can choose between Bowtie (for ungapped alignment) or Bowtie2 (for gapped alignment). In general, Bowtie2 is faster, more sensitive, and memory efficient for reads longer than 50 bp, whereas Bowtie sometimes performs better for relatively short reads (less than 50 bp) (Langmead and Salzberg, 2012). The default parameters for these programs produce satisfactory alignment results, but the user can change them if they wish to achieve a higher alignment rate. The output alignment file is initially saved as a sequence alignment/map (SAM) format and then converted into a sorted BAM (binary version of SAM) format to save storage space and to speed up subsequent data processing.

#### Alignment Filtering

In the ATAC-seq assay, many of the aligned reads cannot be used for downstream data analysis due to mitochondrial DNA contamination, duplicates, and alignment in the blacklist regions of the genome (locations in the genome that tends to show artificially high read signals) (Consortium, 2012). In GUAVA, duplicate reads are removed by Picard MarkDuplicates^3^, while reads aligning to the mitochondria and/or other chromosomes (if required) and blacklist regions are filtered using SAMtools (Li et al., 2009). Finally, only reads which are aligned in proper pair and passed alignment filtering are retained for further processing.

#### Alignment Shifting

When the Tn5 transposase cuts open chromatin regions it introduces two cuts that are separated by 9 bp (Buenrostro et al., 2013). Therefore, ATAC-seq reads aligning to the positive and negative strand need to be adjusted by +4 bp and -5 bp, respectively, to represent the center of the transposase binding site (Buenrostro et al., 2013). In GUAVA, we have included an in-house program to perform the alignment shifting. The output file (a sorted BAM file) from this step is saved with the suffix “ATACseq.bam.” Users can use the alignment shifted bam file as an input bam file for differential analysis.

#### Fragment Size Distribution Graph

Tn5 transposase cuts open chromatin regions and also linker DNA. Therefore, the fragment size distribution graph of a good quality ATAC-seq library has two sharp peaks at <100 bp (open chromatin) and ∼200 bp (mono-nucleosome) and smaller peaks representing di-nucleosomes and tri-nucleosomes. GUAVA uses Picard CollectInsertSizeMetrics^3^ to compute the fragment sizes on alignment shifted bam files and R to plot fragment size distribution graphs.

#### Peak Calling

To identify enriched chromatin regions in ATAC-seq libraries, MACS2 is used for peak calling with the parameters nomodel and nolambda. For differential analysis, users can use the narrowPeak file from MACS2 output.

#### Annotation and Functional Analysis

Graphical User interface for the Analysis and Visualization of ATAC-seq data uses the ChIPpeakAnno (Zhu et al., 2010) bioconductor package for annotation and functional analysis. First, ATAC-seq peaks are categorized into different groups based on their genomic location (i.e., promoter, untranslated regions (UTRs), intron, and exon). Second, peaks that are within 5 kb upstream and 3 kb downstream of the TSS are associated to the nearest genes. Finally, these genes are then analyzed for over-represented gene ontology terms and KEGG pathways (Kanehisa et al., 2017) using ChIPpeakAnno’s getEnrichedGO and getEnrichedPATH functions, respectively.

### Part 2: ATAC-seq Differential Analysis

To use the ATAC-seq differential analysis program in GUAVA, users need to load the processed (filtered and shifted) ATAC-seq alignment bam file and narrowPeak file (hereafter called peak file) for each sample (**Figure 2C**). These files can be found in the output folder of the “ATAC-seq data analysis” program. Next, the user selects the “Next” button to set the fold change and *p*-value cut-off as well as select the output folder to store the results (**Figure 2D**). Finally, the user selects the “start” button to compare ATAC-seq signal from the two conditions. GUAVA will perform the tasks as described below to identify and annotate differentially enriched ATAC-seq peaks. Results such as the differentially enriched peaks, a volcano plot showing gained-open and gained-closed regions between two conditions, over-represented gene ontology and pathways are displayed in the output interface of the “ATAC-seq differential analysis” program (Supplementary Figure S2).

#### Generation of Unique Peak List and Read Count Matrix

To prepare the input data for differential analysis, overlapping peaks from biological replicates are first merged to create a peak list for each condition (e.g., control and treatment). Next, the list of peaks from each condition are then combined to create a final list of unique peaks in which differential analysis is performed. GUAVA then creates a read count matrix by counting the reads in each unique peak for each input sample. To achieve this, GUAVA uses the Rsubread bioconductor package (Liao et al., 2013).

#### Differential ATAC-seq Analysis

To identify differentially enriched ATAC-seq regions, GUAVA uses the DESeq2 bioconductor package. Users can set the fold change and *p*-value cut-off for defining the chromatin accessibility gained (gained-open) and reduced (gained-closed) peaks. Results are displayed in a tabular format in the GUAVA GUI as list of differentially enriched peaks and a volcano plot summarizing the significantly differentially enriched regions.

#### Functional Analysis of Differentially Enriched Peaks

To perform functional analysis on the differentially enriched peaks, they are first categorized into different groups based on their genomic location (i.e., promoter, UTRs, intron, and exon). Next, differentially enriched peaks are annotated and associated to the nearest genes if they occur within the user defined distance from the TSS. Finally, to identify over-represented gene ontology terms and KEGG pathways, GUAVA uses the ChIPpeakAnno bioconductor package.

#### Normalization of ATAC-seq Signals for Visualization on the Integrated Genome Viewer

It is important to normalize ATAC-seq data before visualizing them on the IGV (Thorvaldsdottir et al., 2013) because most likely each sample was sequenced at different depths. Hence, the raw signals are not comparable. GUAVA uses the following method to produce normalized ATAC-seq data tracks. ATAC-seq signals are normalized as reads per million (RPM) so that they can be compared with other biological samples. Normalized data tracks are saved in bigwig format. Output interface of GUAVA allows users to easily view these normalized ATAC-seq signals on the IGV. With one click, the ATAC-seq data track, peaks and genome will be loaded onto the IGV. Multi-threading is used to open multiple instances on the IGV.

## Results and Discussion

In this work, we have developed GUAVA, a fully automated GUI tool, to help researchers, in particular biologists with no computational knowledge, the ability to process and analyze ATAC-seq data on their own. To illustrate the use of GUAVA, we processed and analyzed an ATAC-seq dataset (accession number GSE84515) that was derived from a study examining chromatin accessibility changes in breast cancer cells in response to PI3K inhibitors (Toska et al., 2017). Specifically, ATAC-seq was performed on T47D cells that were treated with either DMSO (vehicle) or the PI3K inhibitor, BYL719. Overall, this dataset consists of four samples with two replicates for each condition (Supplementary Table S1).

### Case Study Demonstrating GUAVA

We first processed the four ATAC-seq samples individually using the ATAC-seq data analysis program. We selected the adapter trimming option with default parameters to remove the sequencing adapters from the raw reads. For read mapping, we used Bowtie2 and the hg19 genome a 2,000 bp insert size and a minimum mapping quality of 30. Since the test dataset is from breast cancer cells, we filtered reads aligning to the Y chromosome as well as the mitochondria by using the “show chromosome” button. For peak calling, a 0.001 *q*-value cut-off was used.

In general, the average mapping rate of the four samples that we processed was >75%. Moreover, over half of the mapped reads passed alignment filtering. The detailed alignment and alignment filtering results for the four samples can be found in Supplementary Table S2. We also observed a fragment size distribution interval of approximately 200 bp for the ATAC-seq peaks representing mono-, di-, and tri-nucleosomes (Supplementary Figure S3). This result indicates the ATAC-seq libraries were of good quality. The ATAC-seq peaks for all the samples were mainly located in the promoter, intergenic, and intronic regions (Supplementary Figure S4). Gene ontology analysis of the individual ATAC-seq samples showed over-represented terms for cell cycle, DNA repair, and the apoptotic signaling pathway (Supplementary Table S3). In pathway analysis of the individual ATAC-seq samples, control samples showed over-represented terms for DNA replication, RNA transport, cell cycle, pathways in cancer, and signaling pathways, while BYL719 treated samples were over-represented in terms for ErbB signaling pathways, p53 signaling pathway, and phosphatidylinositol signaling system (Supplementary Table S4).

**FIGURE 4:**
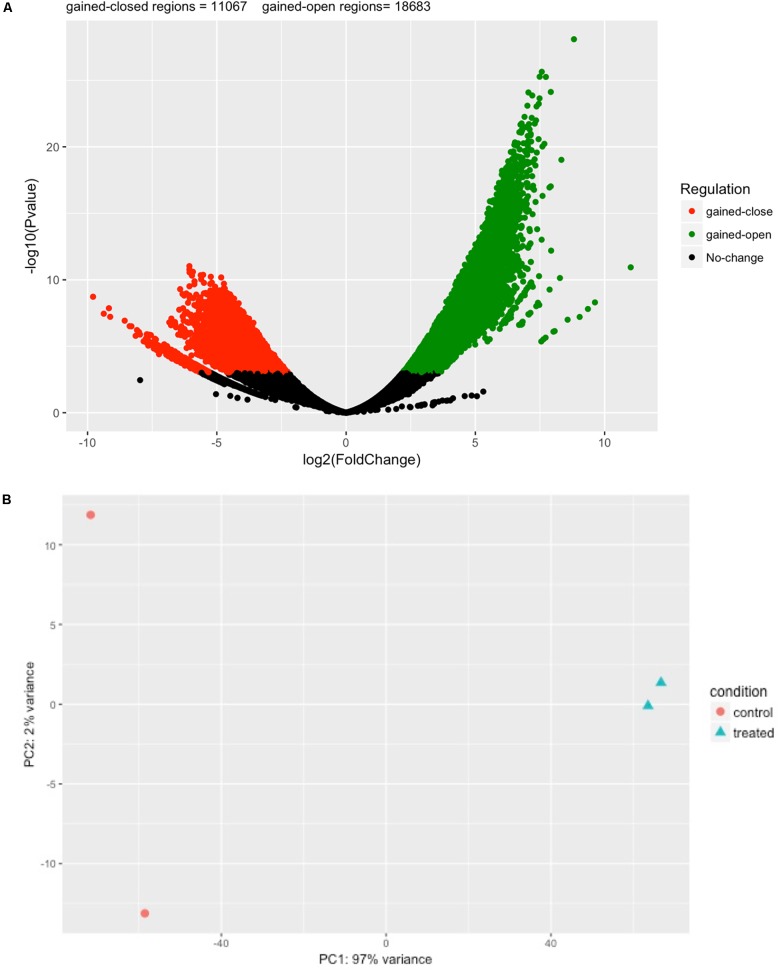
BYL719 induces differentially enriched ATAC-seq peaks in T47D cells. **(A)** A volcano plot showing the differentially enriched peaks between BLY719 and DMSO-treated T47D cells. In total, 29,750 regions were found with differentially enriched ATAC-seq signals. Out of which 18,683 regions were gained-open (increased in chromatin accessibility) and 11,067 were gained-closed regions (decreased in chromatin accessibility) before and after BLY719 treatment of T47D cells. **(B)** Principal component analysis plot for the control and treatment replicates.

**FIGURE 5:**
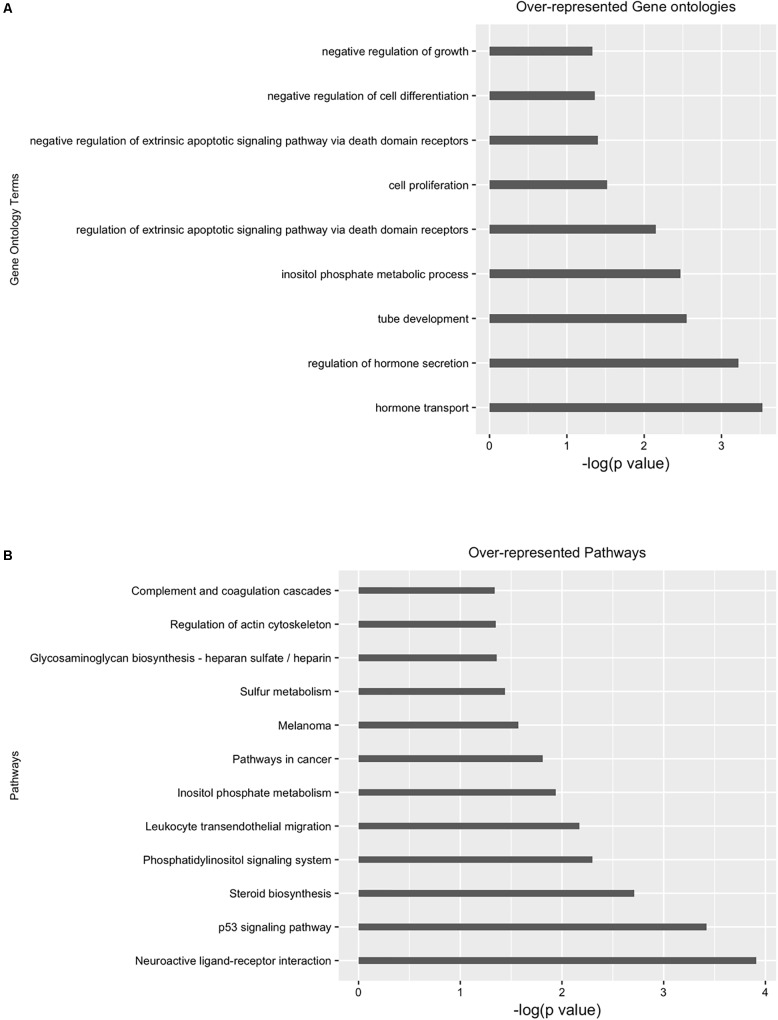
Gene ontology (GO) and pathway analysis of differential enriched peaks. Differentially enriched peaks from **Figure 4** were annotated with nearest downstream genes. Then these genes are used to perform **(A)** gene ontology and **(B)** pathway analysis.

Next, we performed differential analysis on the above processed samples with GUAVA’s ATAC-seq differential analysis program using a twofold change and a 0.001 *p*-value cut-off. For annotation and functional analysis, we used a 5 kb upstream and a 3 kb downstream region for associating peaks with genes. Overall, we identified 11,067 chromatin accessibility locations that were reduced (gained closed) and 18,683 locations with increased chromatin accessibility (gained open) signals upon BYL719 treatment (**Figure 4A**). However, the reproducibility between the replicates in the control samples was less as compared with the replicates of the BYL719 treated samples (**Figure 4B**). Consistent with the known biological effects of BYL719, gene ontology analysis of the differential peaks showed over-represented terms for regulation of cell proliferation, inositol phosphate metabolic process, negative regulation of extrinsic apoptotic signaling pathway, and regulation of extrinsic apoptotic signaling pathway via death domain receptors (**Figure 5A** and Supplementary Data Sheet S2). Similarly, in pathway enrichment analysis, the changes in chromatin accessibility induced by BYL719 associated with processes such as inositol phosphate metabolism, phosphatidylinositol signaling system, p53 signaling pathway, and pathways in cancer (**Figure 5B** and Supplementary Data Sheet S2).

Here, we have presented GUAVA, a simple fully automated GUI software for ATAC-seq analysis. Our main objective behind developing GUAVA was to enable non-programming scientist to analyze ATAC-seq data immediately with no learning curve. The graphs and tables generated by GUAVA will help users to interpret and understand their data. We have created GUAVA with an easy-to-use interface that not only enables users to analyze their ATAC-seq data but also help them to visualize their results. Therefore, GUAVA is a useful user-friendly time saving tool for ATAC-seq analysis. The current version of GUAVA does not perform nucleosome positioning or TF foot-printing analyses and thus, one of our future goals for improving GUAVA will be to include both types of analyses. In addition, future versions of GUAVA will also incorporate modules for assessment of reproducibility of the ATAC-seq signals between the replicates.

### Availability and Future Directions

Graphical User interface for the Analysis and Visualization of ATAC-seq data, together with a complete reference manual, is available at GitHub^4^ and the source code of GUAVA^5^. The sample data and documentation^6^ is available for the demonstration of GUAVA. Furthermore, a video guide ^7^ is available.

Besides incorporating the additional features as mentioned above, we will also continue to make GUAVA as user-friendly as possible by improving the GUI as well as including modules for batch processing samples, and additional differential analysis methods.

## Author Contributions

MD and EC conceived of the described tool. MD contributed to the source code and performed the analyses. ED made intellectual contributions to the development and improvement of the tool. MD and EC contributed to the writing of the manuscript and the online manual.

## Conflict of Interest Statement

The authors declare that the research was conducted in the absence of any commercial or financial relationships that could be construed as a potential conflict of interest.

## Footnotes

1 https://github.com/kundajelab/atac_dnase_pipelines

2 http://www.bioinformatics.babraham.ac.uk/projects/fastqc/

3 http://broadinstitute.github.io/picard

4 https://github.com/MayurDivate/GUAVA

5 https://github.com/MayurDivate/GUAVASourceCode

6 http://ec2-52-201-246-161.compute-1.amazonaws.com/guava/

7 https://www.youtube.com/watch?v=C-9URYWSquI&list=PLBamWP04PQWLGKDdby54LprzSW7jHULSH

## References

[B1] Ahmed Z. Ucar D. (2017). I-ATAC: interactive pipeline for the management and pre-processing of ATAC-seq samples. *PeerJ* 5:e4040. 10.7717/peerj.4040 29181276 PMC5702251

[B2] Buenrostro J. D. Giresi P. G. Zaba L. C. Chang H. Y. Greenleaf W. J. (2013). Transposition of native chromatin for fast and sensitive epigenomic profiling of open chromatin, DNA-binding proteins and nucleosome position. *Nat. Methods* 10 1213–1218. 10.1038/nmeth.2688 24097267 PMC3959825

[B3] Consortium E. P. (2012). An integrated encyclopedia of DNA elements in the human genome. *Nature* 489 57–74. 10.1038/nature11247 22955616 PMC3439153

[B4] Feng J. Liu T. Qin B. Zhang Y. Liu X. S. (2012). Identifying ChIP-seq enrichment using MACS. *Nat. Protoc.* 7 1728–1740. 10.1038/nprot.2012.101 22936215 PMC3868217

[B5] Giresi P. G. Lieb J. D. (2009). Isolation of active regulatory elements from eukaryotic chromatin using FAIRE (Formaldehyde Assisted Isolation of Regulatory Elements). *Methods* 48 233–239. 10.1016/j.ymeth.2009.03.003 19303047 PMC2710428

[B6] Kanehisa M. Furumichi M. Tanabe M. Sato Y. Morishima K. (2017). KEGG: new perspectives on genomes, pathways, diseases and drugs. *Nucleic Acids Res.* 45 D353–D361. 10.1093/nar/gkw1092 27899662 PMC5210567

[B7] Langmead B. Salzberg S. L. (2012). Fast gapped-read alignment with Bowtie 2. *Nat. Methods* 9 357–359. 10.1038/nmeth.1923 22388286 PMC3322381

[B8] Langmead B. Trapnell C. Pop M. Salzberg S. L. (2009). Ultrafast and memory-efficient alignment of short DNA sequences to the human genome. *Genome Biol.* 10:R25. 10.1186/gb-2009-10-3-r25 19261174 PMC2690996

[B9] Li H. Handsaker B. Wysoker A. Fennell T. Ruan J. Homer N. (2009). The sequence alignment/map format and SAMtools. *Bioinformatics* 25 2078–2079. 10.1093/bioinformatics/btp352 19505943 PMC2723002

[B10] Liao Y. Smyth G. K. Shi W. (2013). The subread aligner: fast, accurate and scalable read mapping by seed-and-vote. *Nucleic Acids Res.* 41:e108. 10.1093/nar/gkt214 23558742 PMC3664803

[B11] Love M. I. Huber W. Anders S. (2014). Moderated estimation of fold change and dispersion for RNA-seq data with DESeq2. *Genome Biol.* 15 550. 10.1186/s13059-014-0550-558 25516281 PMC4302049

[B12] Martin M. (2011). Cutadapt removes adapter sequences from high-throughput sequencing reads. *EMBnet J.* 17 10–12. 10.14806/ej.17.1.200

[B13] Ou J. Liu H. Yu J. Kelliher M. A. Castilla L. H. Lawson N. D. (2018). ATACseqQC: a Bioconductor package for post-alignment quality assessment of ATAC-seq data. *BMC Genomics* 19:169. 10.1186/s12864-018-4559-4553 29490630 PMC5831847

[B14] Schep A. N. Buenrostro J. D. Denny S. K. Schwartz K. Sherlock G. Greenleaf W. J. (2015). Structured nucleosome fingerprints enable high-resolution mapping of chromatin architecture within regulatory regions. *Genome Res.* 25 1757–1770. 10.1101/gr.192294.115 26314830 PMC4617971

[B15] Song L. Crawford G. E. (2010). DNase-seq: a high-resolution technique for mapping active gene regulatory elements across the genome from mammalian cells. *Cold Spring Harb. Protoc.* 2010:pdb prot5384. 10.1101/pdb.prot5384 20150147 PMC3627383

[B16] Thorvaldsdottir H. Robinson J. T. Mesirov J. P. (2013). Integrative genomics viewer (IGV): high-performance genomics data visualization and exploration. *Brief. Bioinform.* 14 178–192. 10.1093/bib/bbs017 22517427 PMC3603213

[B17] Toska E. Osmanbeyoglu H. U. Castel P. Chan C. Hendrickson R. C. Elkabets M. (2017). PI3K pathway regulates ER-dependent transcription in breast cancer through the epigenetic regulator KMT2D. *Science* 355 1324–1330. 10.1126/science.aah6893 28336670 PMC5485411

[B18] Zhu L. J. Gazin C. Lawson N. D. Pages H. Lin S. M. Lapointe D. S. (2010). ChIPpeakAnno: a Bioconductor package to annotate ChIP-seq and ChIP-chip data. *BMC Bioinformatics* 11:237. 10.1186/1471-2105-11-237 20459804 PMC3098059

