# Supplementary material for: GUAVA: A Graphical User Interface for the Analysis and Visualization of ATAC-seq Data
Source: Front Genet. 2018 Jul 17;9:250. doi: 10.3389/fgene.2018.00250 (PMC6056626; doi:10.3389/fgene.2018.00250)
Supplement: Supplementary file 1 [file Presentation_1.pdf]

Supplementary Material

GUAVA: a Graphical User interface for the Analysis and Visualization of ATAC-seq data

Mayur Divate, Edwin Cheung*

*** Correspondence:** Corresponding Author: echeung@umac.mo

1 Supplementary Figures and Tables

1.1 Supplementary Figures

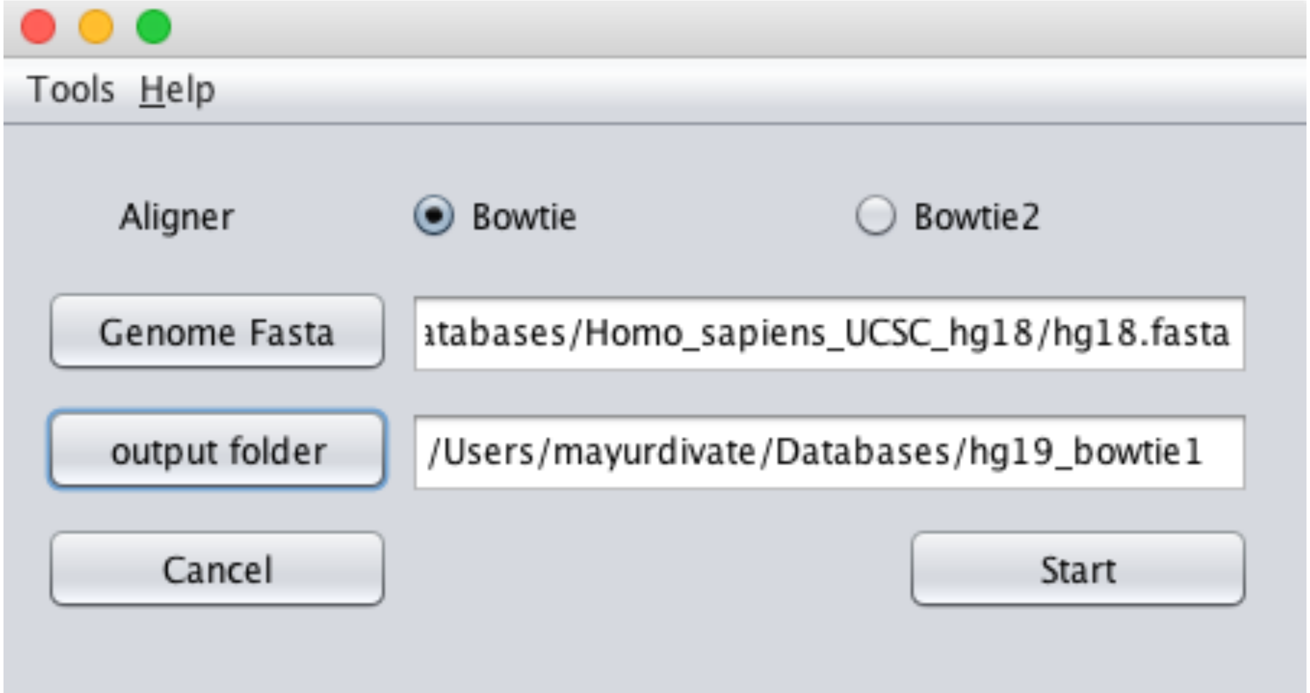

Figure S1. Genome index builder. Input window for the genome index builder program. Here, users choose between Bowtie and Bowtie2. Users also upload the genome fasta file using the 'Genome Fasta' button. Users also select the output folder here. Finally, clicking on the 'Start' button will start the genome index builder program.

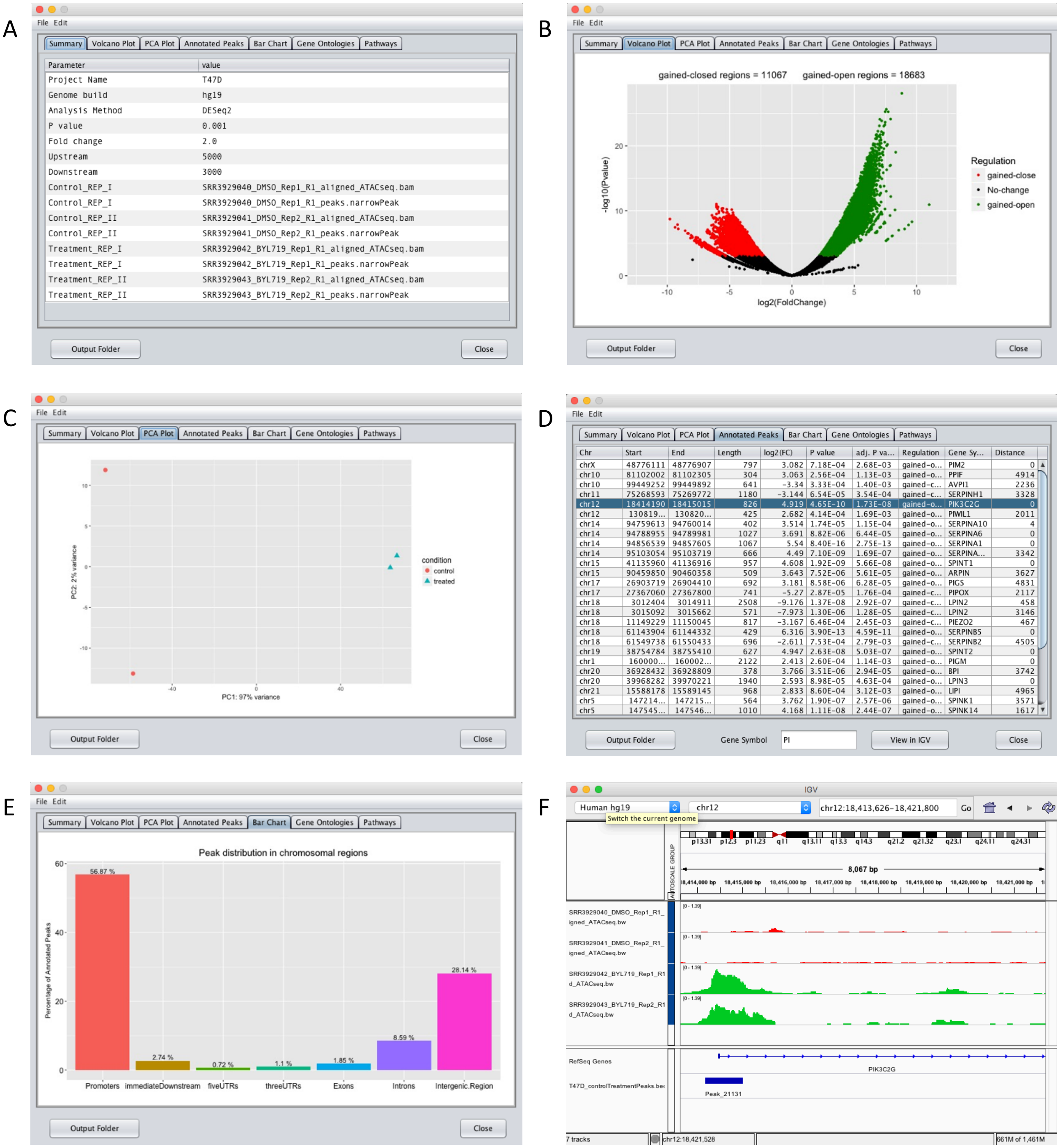

Parameter	value
Project Name	T47D
Genome build	hg19
Analysis Method	DESeq2
P value	0.001
Fold change	2.0
Upstream	5000
Downstream	3000
Control_REP_I	SRR3929040_DMSO_Rep1_R1_aligned_ATACseq.bam
Control_REP_I	SRR3929040_DMSO_Rep1_R1_peaks.narrowPeak
Control_REP_II	SRR3929041_DMSO_Rep2_R1_aligned_ATACseq.bam
Control_REP_II	SRR3929041_DMSO_Rep2_R1_peaks.narrowPeak
Treatment_REP_I	SRR3929042_BYL719_Rep1_R1_aligned_ATACseq.bam
Treatment_REP_I	SRR3929042_BYL719_Rep1_R1_peaks.narrowPeak
Treatment_REP_II	SRR3929043_BYL719_Rep2_R1_aligned_ATACseq.bam
Treatment_REP_II	SRR3929043_BYL719_Rep2_R1_peaks.narrowPeak

Chr	Start	End	Length	log2(FC)	P value	adj. P va...	Regulation	Gene Sy...	Distance
chrX	48776111	48776907	797	3.082	7.18E-04	2.68E-03	gained-o...	PIM2	0
chr10	81102002	81102305	304	3.063	2.56E-04	1.13E-03	gained-o...	PPIF	4914
chr10	99449252	99449892	641	-3.34	3.33E-04	1.40E-03	gained-c...	AVPI1	2236
chr11	75268593	75269772	1180	-3.144	6.54E-05	3.54E-04	gained-c...	SERPINH1	3328
chr12	18414190	18415015	826	4.919	4.65E-10	1.73E-08	gained-o...	PIK3C2G	0
chr12	130819...	130820...	425	2.682	4.14E-04	1.69E-03	gained-o...	PIWIL1	2011
chr14	94759613	94760014	402	3.514	1.74E-05	1.15E-04	gained-o...	SERPINA10	4
chr14	94788955	94789981	1027	3.691	8.82E-06	6.44E-05	gained-o...	SERPINA6	0
chr14	94856539	94857605	1067	5.54	8.40E-16	2.75E-13	gained-o...	SERPINA1	0
chr14	95103054	95103719	666	4.49	7.10E-09	1.69E-07	gained-o...	SERPINA...	3342
chr15	41135960	41136916	957	4.608	1.92E-09	5.66E-08	gained-o...	SPINT1	0
chr15	90459850	90460358	509	3.643	7.52E-06	5.61E-05	gained-o...	ARPIN	3627
chr17	26903719	26904410	692	3.181	8.58E-06	6.28E-05	gained-o...	PIGS	4831
chr17	27367060	27367800	741	-5.27	2.87E-05	1.76E-04	gained-c...	PIPOX	2117
chr18	3012404	3014911	2508	-9.176	1.37E-08	2.92E-07	gained-c...	LPIN2	458
chr18	3015092	3015662	571	-7.973	1.30E-06	1.28E-05	gained-c...	LPIN2	3146
chr18	11149229	11150045	817	-3.167	6.46E-04	2.45E-03	gained-c...	PIEZO2	467
chr18	61143904	61144332	429	6.316	3.90E-13	4.59E-11	gained-o...	SERPINB5	0
chr18	61549738	61550433	696	-2.611	7.53E-04	2.79E-03	gained-c...	SERPINB2	4505
chr19	38754784	38755410	627	4.947	2.63E-08	5.03E-07	gained-o...	SPINT2	0
chr1	160000...	160002...	2122	2.413	2.60E-04	1.14E-03	gained-o...	PIGM	0
chr20	36928432	36928809	378	3.766	3.51E-06	2.94E-05	gained-o...	BPI	3742
chr20	39968282	39970221	1940	2.593	8.98E-05	4.63E-04	gained-o...	LPIN3	0
chr21	15588178	15589145	968	2.833	8.60E-04	3.12E-03	gained-o...	LIPI	4965
chr5	147214...	147215...	564	3.762	1.90E-07	2.57E-06	gained-o...	SPINK1	3571
chr5	147545...	147546...	1010	4.168	1.11E-08	2.44E-07	gained-o...	SPINK14	1617

G

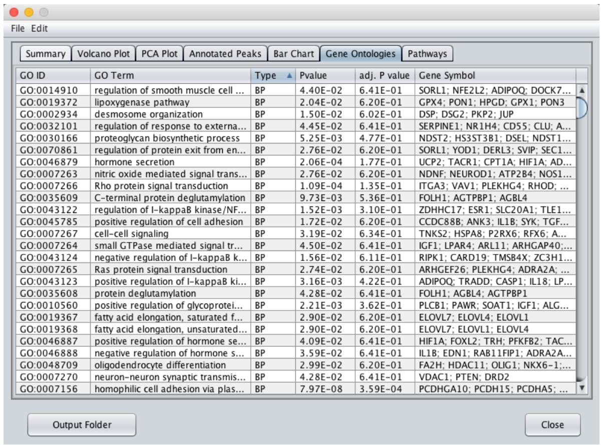

GO ID	GO Term	Type	Pvalue	adj. P value	Gene Symbol
GO:0014910	regulation of smooth muscle cell ...	BP	4.40E-02	6.41E-01	SORL1; NFE2L2; ADIPOQ; DOCK7...
GO:0019372	lipoxygenase pathway	BP	2.04E-02	6.20E-01	GPX4; PON1; HPGD; GPX1; PON3
GO:0002934	desmosome organization	BP	1.50E-02	6.02E-01	DSP; DSG2; PKP2; JUP
GO:0032101	regulation of response to externa...	BP	4.45E-02	6.41E-01	SERPINE1; NR1H4; CD55; CLU; A...
GO:0030166	proteoglycan biosynthetic process	BP	5.25E-03	4.77E-01	NDST2; HS3ST3B1; DSEL; NDST1...
GO:0070861	regulation of protein exit from en...	BP	2.76E-02	6.20E-01	SORL1; YOD1; DERL3; SVIP; SEC1...
GO:0046879	hormone secretion	BP	2.06E-04	1.77E-01	UCP2; TACR1; CPT1A; HIF1A; AD...
GO:0007263	nitric oxide mediated signal trans...	BP	2.76E-02	6.20E-01	NDNF; NEUROD1; ATP2B4; NOS1...
GO:0007266	Rho protein signal transduction	BP	1.09E-04	1.35E-01	ITGA3; VAV1; PLEKHG4; RHOD; ...
GO:0035609	C-terminal protein deglutamylation	BP	9.73E-03	5.36E-01	FOLH1; AGTPBP1; AGBL4
GO:0043122	regulation of I-kappaB kinase/NF...	BP	1.52E-03	3.10E-01	ZDHHC17; ESR1; SLC20A1; TLE1...
GO:0045785	positive regulation of cell adhesion	BP	1.72E-02	6.20E-01	CCDC88B; ANK3; IL1B; SYK; TGF...
GO:0007267	cell-cell signaling	BP	3.19E-02	6.34E-01	TNKS2; HSPA8; P2RX6; RFX6; A...
GO:0007264	small GTPase mediated signal tr...	BP	4.50E-02	6.41E-01	IGF1; LPAR4; ARL11; ARHGAP40;...
GO:0043124	negative regulation of I-kappaB k...	BP	1.56E-02	6.11E-01	RIPK1; CARD19; TMSB4X; ZC3H1...
GO:0007265	Ras protein signal transduction	BP	2.74E-02	6.20E-01	ARHGEF26; PLEKHG4; ADRA2A; ...
GO:0043123	positive regulation of I-kappaB ki...	BP	3.16E-03	4.22E-01	ADIPOQ; TRADD; CASP1; IL18; LP...
GO:0035608	protein deglutamylation	BP	4.28E-02	6.41E-01	FOLH1; AGBL4; AGTPBP1
GO:0010560	positive regulation of glycoprotei...	BP	2.21E-03	3.62E-01	PLCB1; PAWR; SOAT1; IGF1; ALG...
GO:0019367	fatty acid elongation, saturated f...	BP	2.90E-02	6.20E-01	ELOVL7; ELOVL4; ELOVL1
GO:0019368	fatty acid elongation, unsaturated...	BP	2.90E-02	6.20E-01	ELOVL7; ELOVL1; ELOVL4
GO:0046887	positive regulation of hormone se...	BP	4.09E-02	6.41E-01	HIF1A; FOXL2; TRH; PFKFB2; TAC...
GO:0046888	negative regulation of hormone s...	BP	3.59E-02	6.41E-01	IL1B; EDN1; RAB11FIP1; ADRA2A...
GO:0048709	oligodendrocyte differentiation	BP	2.99E-02	6.20E-01	FA2H; HDAC11; OLIG1; NKX6-1;...
GO:0007270	neuron-neuron synaptic transmis...	BP	4.28E-02	6.41E-01	VDAC1; PTEN; DRD2
GO:0007156	homophilic cell adhesion via plas...	BP	7.97E-08	3.59E-04	PCDHGA10; PCDH15; PCDHA5; ...

H

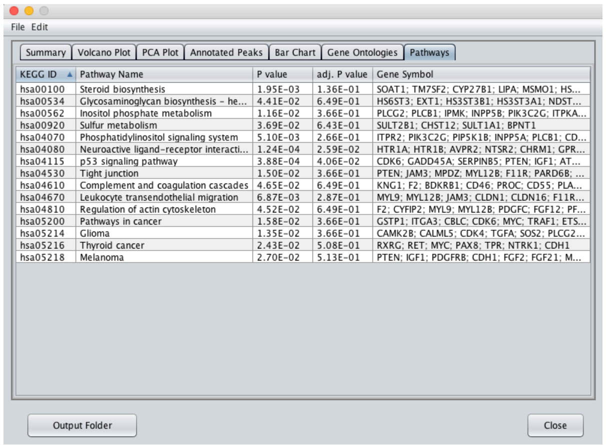

KEGG ID	Pathway Name	P value	adj. P value	Gene Symbol
hsa00100	Steroid biosynthesis	1.95E-03	1.36E-01	SOAT1; TM7SF2; CYP27B1; LIPA; MSMO1; HS...
hsa00534	Glycosaminoglycan biosynthesis - he...	4.41E-02	6.49E-01	HS6ST3; EXT1; HS3ST3B1; HS3ST3A1; NDST...
hsa00562	Inositol phosphate metabolism	1.16E-02	3.66E-01	PLCG2; PLCB1; IPMK; INPP5B; PIK3C2G; ITPKA...
hsa00920	Sulfur metabolism	3.69E-02	6.43E-01	SULT2B1; CHST12; SULT1A1; BPNT1
hsa04070	Phosphatidylinositol signaling system	5.10E-03	2.66E-01	ITPR2; PIK3C2G; PIP5K1B; INPP5A; PLCB1; CD...
hsa04080	Neuroactive ligand-receptor interacti...	1.24E-04	2.59E-02	HTR1A; HTR1B; AVPR2; NTSR2; CHRM1; GPR...
hsa04115	p53 signaling pathway	3.88E-04	4.06E-02	CDK6; GADD45A; SERPINB5; PTEN; IGF1; AT...
hsa04530	Tight junction	1.50E-02	3.66E-01	PTEN; JAM3; MPDZ; MYL12B; F11R; PARD6B; ...
hsa04610	Complement and coagulation cascades	4.65E-02	6.49E-01	KNG1; F2; BDKRB1; CD46; PROC; CD55; PLA...
hsa04670	Leukocyte transendothelial migration	6.87E-03	2.87E-01	MYL9; MYL12B; JAM3; CLDN1; CLDN16; F11R...
hsa04810	Regulation of actin cytoskeleton	4.52E-02	6.49E-01	F2; CYFIP2; MYL9; MYL12B; PDGFC; FGF12; PF...
hsa05200	Pathways in cancer	1.58E-02	3.66E-01	GSTP1; ITGA3; CBLC; CDK6; MYC; TRAF1; ETS...
hsa05214	Glioma	1.35E-02	3.66E-01	CAMK2B; CALML5; CDK4; TGFA; SOS2; PLCG2...
hsa05216	Thyroid cancer	2.43E-02	5.08E-01	RXRG; RET; MYC; PAX8; TPR; NTRK1; CDH1
hsa05218	Melanoma	2.70E-02	5.13E-01	PTEN; IGF1; PDGFRB; CDH1; FGF2; FGF21; M...

Figure S2. Output interface for GUAVA ATAC-seq differential analysis. A) Input summary. B) Volcano plot indicating the differentially enriched peaks. Red: peaks with increased chromatin accessibility, green: peaks with reduced chromatin accessibility and black: peaks with no significant change in chromatin accessibility. C) Principal component analysis (PCA) plot for the control and treated replicates D) A table containing the annotated differentially enriched ATAC-seq peaks. This tab also provides easy access to IGV to visualize differentially enriched peaks and normalized ATAC-seq signals for each sample E) Bar chart showing the percentage of peaks in various genomic locations such as the promoter, intron, exon, UTR, etc. F) Peak visualization in IGV. G) Over represented gene ontologies and H) KEGG pathways

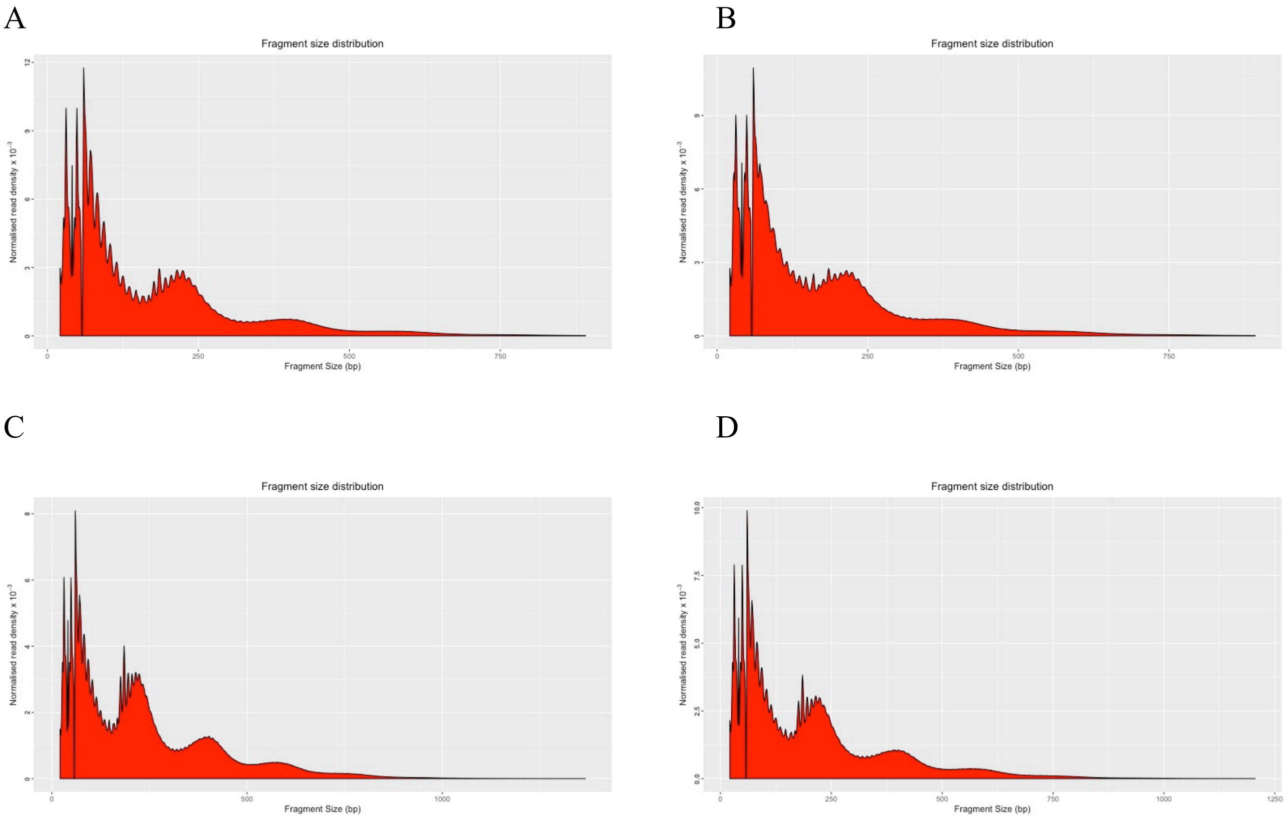

Figure S3. Fragment size distribution graphs. Graphs showing the fragment size distribution of ATAC-seq reads in T47D cells for A) DMSO replicate 1, B) DMSO replicate 2, C) BYL719 replicate 1, and D) BYL719 replicate 2.

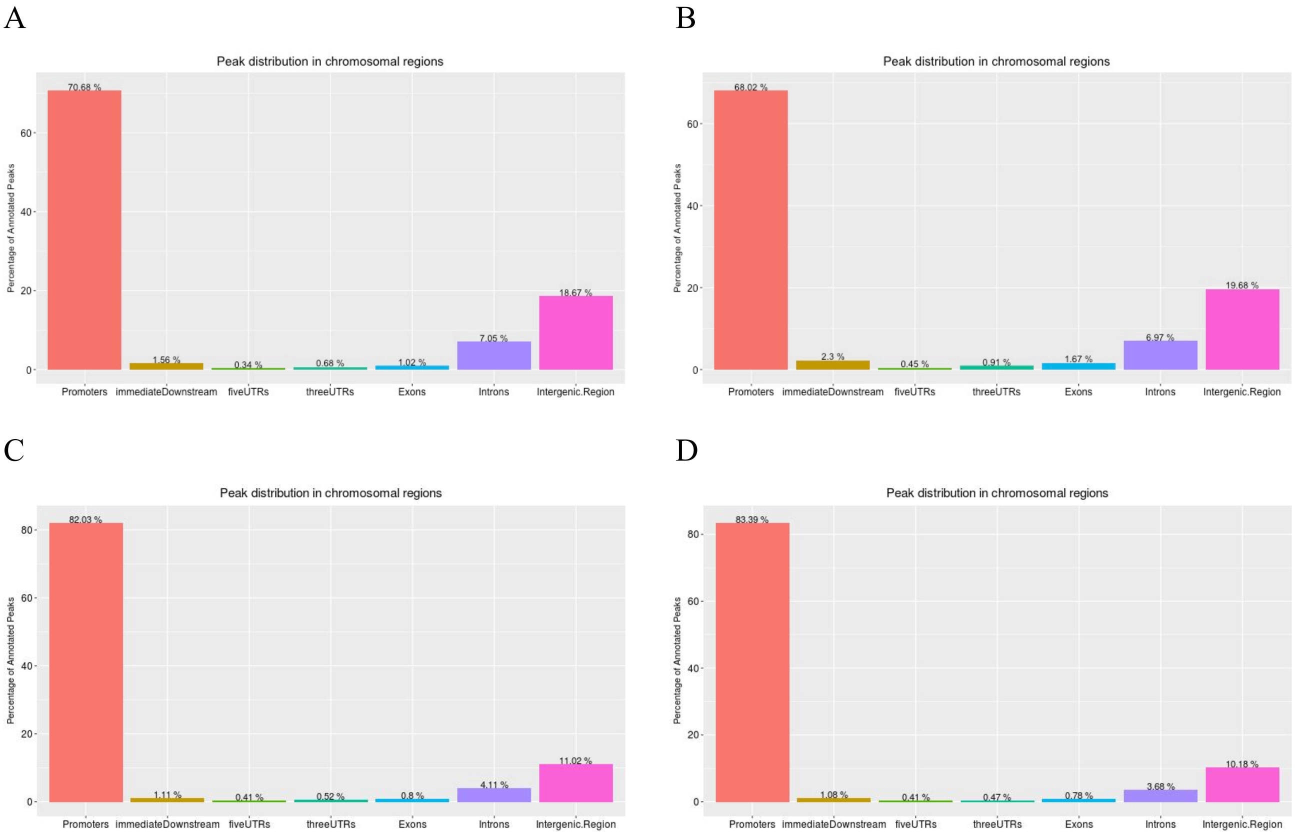

Figure S4. Genomic distribution of ATAC-seq peaks. Bar charts showing the distribution of ATAC-seq peaks for A) DMSO replicate 1, B) DMSO replicate 2, C) BYL719 replicate 1, and D) BYL719 replicate 2.

1.2 Supplementary Tables

Table S1. GEO accession number for the samples from the case study.

Sample	GEO Accession Number	Treatment	Replicate Number
DMSO_Rep1	GSM2241147	DMSO	Replicate I
DMSO_Rep2	GSM2241148	DMSO	Replicate II
BYL719_Rep1	GSM2241149	BYL719	Replicate I
BYL719_Rep2	GSM2241150	BYL719	Replicate II

Table S2. GUAVA alignment and peak calling statistics.

Sample Name	BYL719_Rep1	BYL719_Rep2	DMSO_Rep1	DMSO_Rep2
Total Reads	44892526	29411509	101692391	94208170
Total number of reads aligned	34410274 (76.65%)	22322001 (75.9%)	82027622 (80.66%)	75771166 (80.43%)
Mapping quality filtered reads	7551956 (16.82%)	5059044 (17.2%)	13940905 (13.71%)	10717722 (11.38%)
Total number of reads failed to align	2930296 (6.53%)	2030464 (6.9%)	5723864 (5.63%)	7719282 (8.19%)
Reads Mapped to ChrM	7957443 (17.73%)	5496204 (18.69%)	15552430 (15.3%)	2907706 (3.09%)
Duplicate Reads	12865311 (28.66%)	7001849 (23.81%)	25430014 (25.01%)	13855635 (14.71%)
Total Useful Reads	19952669 (44.45%)	14051507 (47.78%)	55033289 (54.12%)	60956787 (64.7%)
Total number of Peaks identified	65735	56734	151009	147675
